# Association between ABCB1 (3435C>T) polymorphism and susceptibility of colorectal cancer: A meta-analysis

**DOI:** 10.1097/MD.0000000000019189

**Published:** 2020-02-21

**Authors:** Li-li Han, Bai-le Zuo, Wei-liang Cai, Zhen-ni Guo, Bing-hua Tong, Hui-lian Wei, Zheng Zhu, Guo-yin Li

**Affiliations:** a College of Life Science and Agronomy, Zhoukou Normal University; b Department of Respiratory, Zhoukou Central Hospital, Zhoukou; c Tumor Molecular Immunology and Immunotherapy Laboratory, School of Laboratory Medicine, Xinxiang Medical University, Xinxiang; d Department of Orthopedics Surgery, The Second Xiangya Hospital of Central South University, Changsha, Hunan, China; e Department of Internal Medicine, Division of Hematology/Oncology, University of California Davis, Sacramento, CA, USA.

**Keywords:** ABCB1 gene, colorectal cancer, meta-analysis, polymorphism, susceptibility

## Abstract

Studies on the relationship between ABCB1 3435C>T polymorphism (rs1045642) and colorectal cancer (CRC)susceptibility have yielded inconclusive results. To clarify this issue, we undertook a meta-analysis to investigate the relationship between rs1045642 and CRC risk.

Three electronic scientific publication databases (Cochrane Library, Pubmed, Embase) were screened using specific search terms. Relevant literature was identified using literature traceability methods. Selected publications were evaluated according to the inclusion and exclusion criteria. Effect size information (odds ratio and the corresponding 95% confidence interval [CI]) was obtained following quality assessment and data extraction from the included publications, and a meta-analysis conducted. Statistical analysis was performed with the Stata sofz (Version 13.0) software.

Overall, 17 case-control studies involving 7129 CRC patients and 7710 healthy control subjects satisfied the criteria for inclusion in the meta-analysis. There was no significant association between ABCB1 3435C>T polymorphism and CRC risk in any of the genetic models. In the CC versus CT model (*I*^*2*^ = 20.9%, *P*_heterogeneity_ = .276), CC versus CT + TT model (*I*^*2*^ = 45.6%, *P*_heterogeneity_ = .102) and CT versus CC + TT model (*I*^*2*^ = 17.8%, *P*_heterogeneity_ = .298) analyses, between-study heterogeneities were detected as significant in Asian populations. In the CT versus TT model (*I*^*2*^ = 24%, *P*_heterogeneity_ = .254) and CC + CT versus TT model (*I*^*2*^ = 0, *P*_heterogeneity_ = .55), between-study heterogeneities were found to be significant in groups of different populations.

The meta-analysis described here suggests that the ABCB1 3435C>T polymorphism is not related to CRC susceptibility.

## Abstract 2

Supplemental Digital Content is available in the text

## 1 Introduction

In a global context, colorectal cancer (CRC) represents a serious threat to human life and health. What is more, it confers an enormous economic burden on society. CRC is the third most commonly diagnosed cancer in the United States. Its estimated morbidity and mortality incidence will be the third among all carcinomas in the United States in 2019.^[1]^ The incidence and mortality rates of CRC vary substantially by race/ethnicity.^[2]^ Lifestyle difference is also a vital factor leading to the striking variation in CRC morbidity globally.^[3]^ Differences in access to prevention, the quality of treatment technology, and the economic level of the patients greatly affect the mortality rate of CRC patients.^[4]^ Although the incidence of CRC in the United States has been tapering off in recent years, worldwide, the outlook does not give grounds for optimism.^[1,5,6]^

The adenosine triphosphate-binding cassette subfamily B member 1 (ABCB1) gene, also known as multidrug resistance gene 1 (MDR1), is located on the chromosomal region 7q21.1 and encodes the P-glycoprotein (P-gp). P-gp is a 1280-amino acid transporter that serves as a genetically polymorphic efflux transporter that removes foreign substances from cells.^[7]^ P-gp mediates multiple drug resistance in cancer cells through various signaling pathways, such as the cyclic adenosine monophosphate/protein kinase A pathway,^[8,9]^ the phosphatidylinositol 3-kinase/protein kinase B pathway,^[10–12]^ the Y-box binding protein 1,^[13,14]^ the phosphatase and tensin homolog,^[15,16]^ p53,^[17]^ protein kinase C,^[18]^ and other protein kinases.^[19]^ Previous studies have shown that ABCB1 is overexpressed in a variety of tumors, such as breast cancer, acute myeloid leukemia, hematological malignancies, childhood tumors, and other solid tumors.^[20]^

Single nucleotide polymorphisms (SNPs) of ABCB1 affect its expression and function.^[21]^ Up to now, numerous SNPs, including some synonymous ones, have been identified in the coding region.^[21]^ Three SNPs in the coding sequence (rs1128503, rs1045642, and rs2032582) are the most widely studied in ABCB1; these are relevant to the substrate and inhibitor-dependent functional modifications observed in vitro and reduced expression in tissues.^[21]^ The distributions of rs1128503, rs1045642, and rs2032582 differ significantly among races and ethnicities. It is reported that Africans and African-Americans harbor the lowest frequencies of polymorphic alleles and Asians and Caucasians possess the highest.^[21]^

The ABCB1 3435C>T polymorphism is a synonymous SNP with no impact on the structural contribution of the amino acid at position 1145 (Ile) in the second ATP binding domain but does affect the expression of P-gp in tissues.^[22]^ So far, numerous epidemiological studies have been performed to assess the association between rs1045642 and risk for CRC. However, due to the limitations of individual studies, the results are inconsistent. To help resolve this matter, we performed a meta-analysis based on a total of 17 independent studies, to obtain a more precise estimation of the association between rs1045642 and the risk of CRC. This meta-analysis suggested ABCB1 3435C>T polymorphism is not related to CRC susceptibility.

## 2 Materials and methods

### 2.1 Literature search

We queried the Cochrane Library, Pubmed, and Embase databases on August 1, 2019. Keyword combinations for colorectal neoplasms (colorectal, colorectal tumor, colorectal neoplasm, colorectal tumors, tumor colorectal, neoplasms colorectal, neoplasm colorectal, cancers colorectal, cancer colorectal, CRC, carcinomas colorectal, colorectal carcinoma, carcinoma colorectal, colorectal carcinomas) or colonic neoplasms (colonic neoplasm; colon neoplasm; neoplasms colonic, neoplasm colon, colon neoplasms, neoplasms colon, neoplasm colonic, cancer colonic, cancers colon, cancer of the colon, colonic cancer, colon cancer, colon cancers, colonic cancers, cancers colonic, cancer colon, cancer of colon) and gene symbols, and synonyms for the ABCB1 gene (ABCB1, MDR1, CLCS, P-GP, PGY1, ABC20, CD243, and GP170) and polymorphism (polymorphism, SNP, and variant) were used to form a Boolean query formula. Two reviewers (L.H. and Z.Z.), independently and in duplicate, screened titles and abstracts using a standardized data form tested in pilot runs. Inconsistencies regarding inclusion were resolved through consensus. The meta-analysis did not involve data related to patient personal information and therefore does not require ethical approval.

### 2.2 Inclusion and exclusion criteria

The criteria for inclusion in the study were as follows:

(1) manuscripts from peer-reviewed journals;
(2) case-control studies assessing the association between the ABCB1 3435C>T polymorphism (rs1045642) and CRC;
(3) studies focusing on CRC or colonic cancer;
(4) no inconsistencies in genotype data for either cases or controls; and
(5) studies with enough genotype data to estimate the odds ratio (OR) and 95% confidence interval (CI) in at least one genetic comparison model.

The exclusion criteria were:

(1) not case-control studies,
(2) control population including malignant tumor patients, and
(3) duplicate publications.

Two individual authors (L.H. and Z.Z.) performed the literature selection process. Another author (B.Z.) performed an investigation to reach an eventual agreement if the first 2 reviewers came to contradictory conclusions.

### 2.3 Data extraction

Two investigators reviewed and extracted information from all qualified publications based on the inclusion and exclusion criteria listed above. When there was a conflict, the 2 reviewers reached an agreement through discussion. The following information was extracted from each included study: first author's surname, year of publication, ethnicity, total numbers of cases and controls, as well as numbers of cases and controls with CC, CT, and TT genotypes. Individuals of different descent were categorized as Caucasian and Asian. Individuals of different descent were categorized as Caucasian and Asian.

### 2.4 Methodological quality assessment

The quality of the included studies was evaluated by the total score of quality assessment (TSQA).^[23]^ Studies were scored according to TSQA standards (Supplementary Table S1). Studies of high quality were given scores of greater than 9.

### 2.5 Statistical analysis

The measure of effect in these studies was the OR with 95% CI. Summary measures were pooled using random-effects models, with the estimate of heterogeneity taken from the Mantel-Haenszel model. All statistical analyses were conducted in the Stata 13 environment. The aggregated estimate of the OR and corresponding 95% CI were calculated for the dominant model (CC + CT vs TT, with C standing for cytosine and T for thymine), the recessive model (CC vs CT + TT), and the overdominant model (CT vs CC + TT). Cochran's Chi-square-based *Q* test was used to test the heterogeneity assumption. A value of *P* < .1 in the *Q* test indicated that the between-study heterogeneity was significant.^[24]^ However, when *P*≥.1, the pooled ORs and 95% CIs should be measured using a fixed-effect model employing the Mantel–Haenszel algorithm.^[25]^ To explore the effect of heterogeneity among the studies on the conclusions of this meta-analysis, we performed subgroup analyses by ethnicity. We examined the ABCB1 3435C>T genotypes using dominant (CC + CT vs TT), recessive (CC vs CT + TT), and overdominant (CT vs CC + TT) genetic models, as well as the allelic model (C vs T). The estimated OR and 95% CI were obtained from Forest plots. Publication bias was graphically detected by funnel plots. The symmetry of the funnel plot was further evaluated by Egger linear regression test. The significance of the intercept was determined by the *t* test suggested by Egger, where *P* < .05 was considered representative of statistically significant publication bias.

## 3 Results

### 3.1 Retrieval of studies and their characteristics

**Figure 1:**
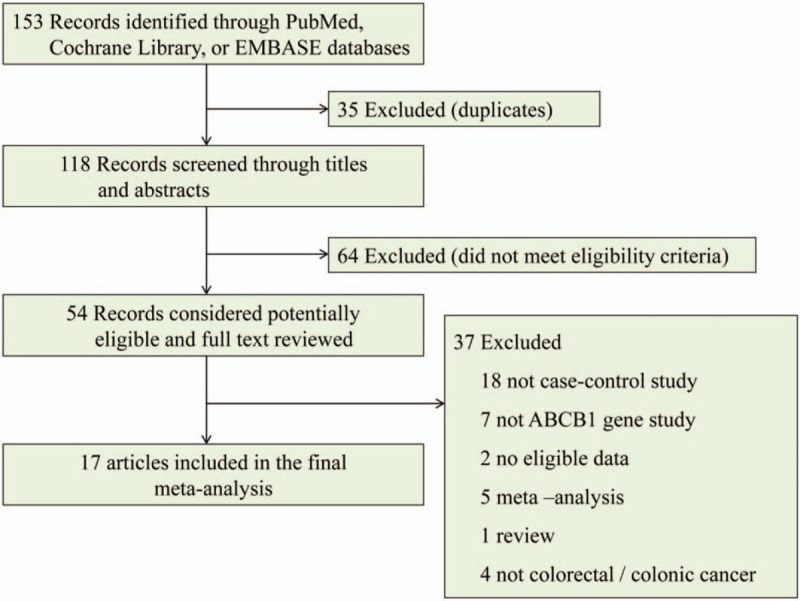
Flow diagram of literature search and screen.

From the searches for studies on colorectal neoplasms or colonic neoplasms and C3435T genotypes, 153 potentially eligible records were identified. Titles and abstracts of these records were screened for inclusion. Seventeen independent studies met the inclusion criteria, consisting of six Asian and eleven Caucasian populations (Fig. 1).^[26–42]^ In total, 7,179 CRC cases and 7,710 controls were included in the meta-analysis. The characteristics of the selected studies are summarized in Table 1.

**Table 1:**
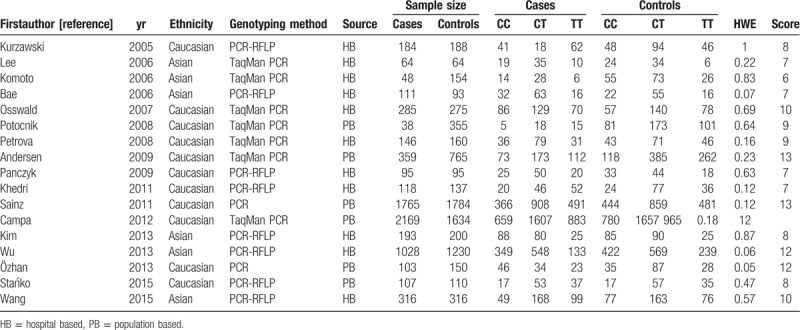
Main characteristics of studies included in this meta-analysis.

### 3.2 Association of rs1045642 C>T and CRC

A total of 17 independent studies consisting of 7129 CRC patients and 7710 healthy controls were included in the analysis of the association of ABCB1 3435C>T polymorphism with susceptibility to CRC. All possible genetic models were analyzed to seek potential differences in genotypic and allelic frequencies regarding ABCB1 3435 C>T polymorphism amongst CRC cases and controls. We used the random effect model for the analysis of all genetic models: we did not find a significant association between the different genotypes of SNP rs1045642 C>T and susceptibility to CRC in any of the models (Table 2; Fig. S1). Moreover, we found no association between ABCB1 3435 C>T polymorphism and CRC when comparing the C and T alleles (Table 2; Fig. S2).

**Table 2:**
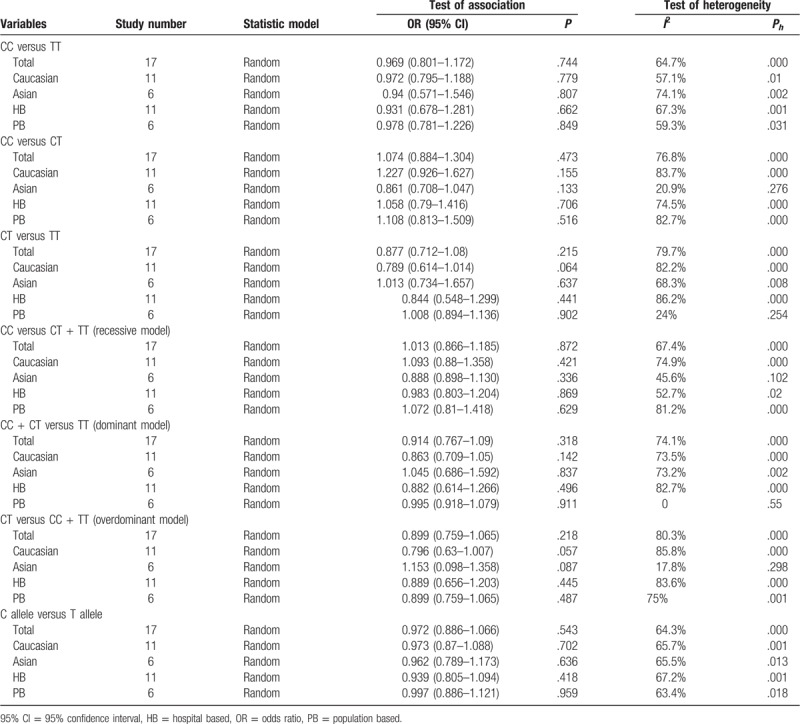
Main results of rs1045642 polymorphism and colorectal cancer risk in this meta-analysis.

### 3.3 Subgroup analysis based on ethnicity

**Figure 2:**
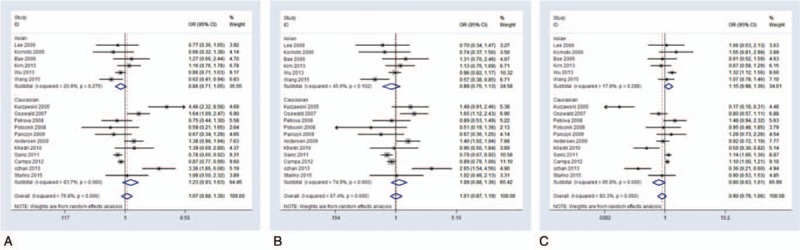
Forest plot on association between rs1045642 polymorphism and colorectal cancer risk, stratified by ethnicity. (A) CC versus CT model, random-effect pooled OR = 1.074, 95% CI: 0.884–1.304, *P* = .473, *I*^*2*^ = 76.8%, *P*_heterogeneity_ = .000. (B) CC versus CT + TT model, random-effect pooled OR = 1.013, 95% CI: 0.866–1.185, *P* = .872, *I*^*2*^ = 67.4%, *P*_heterogeneity_ = .000. **(C)** CT versus CC + TT random-effect pooled OR = 0.899, 95% CI: 0.759–1.065, *P* = .218, *I*^*2*^ = 80.3%, *P*_heterogeneity_ = .000. 95% CI = 95% confidence interval, OR = odds ratio.

**Figure 3:**
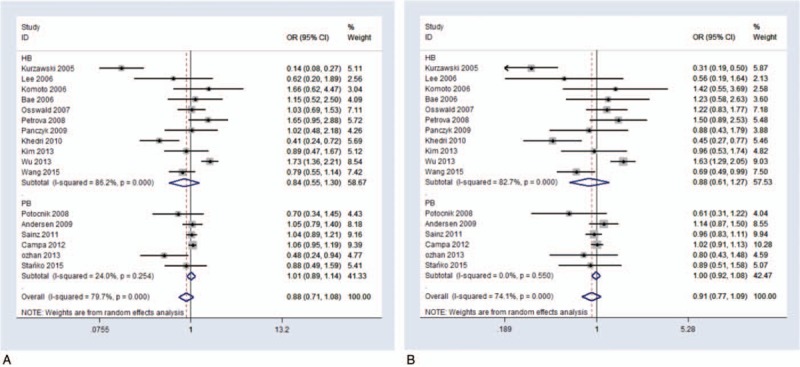
Forest plot on association between rs1045642 polymorphism and colorectal cancer risk, stratified by source. (A) CT versus TT model, random-effect pooled OR = 0.877, 95% CI: 0.712–1.08, *P* = .215, *I*^*2*^ = 79.7%, *P*_heterogeneity_ = .000. (B) CC + CT versus TT model, random-effect pooled OR = 0.914, 95% CI: 0.767–1.09, *P* = .318, *I*^*2*^ = 74.1%, *P*_heterogeneity_ = .000. 95% CI = 95% confidence interval, OR = odds ratio.

**Figure 4:**
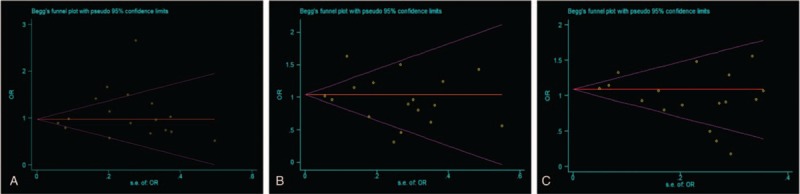
Funnel plot analysis to detect publication bias for rs1045642 polymorphism. (A) Recessive model. (B) Dominant model. (C) Overdominant model. Each point represents a separate study for the indicated association.

In general, between-study heterogeneities were not significant in any of the genetic models for the association between ABCB1 3435C>T polymorphism and CRC, but in the stratified analysis by ethnicity, they were present in some genetic models (Table 2). Eleven studies consisting of 5369 CRC cases and 5653 controls were included in the Caucasian group, while six studies comprising 1760 CRC cases and 2057 controls were enrolled in the Asian group. We used the random-effect model for examining heterogeneity using the genetic models described above. For the Caucasian group, no significant between-study heterogeneity was detected (Figs. 2–4; Fig. S3). For the Asian group, the between-study heterogeneities increased strikingly. In the CC versus CT model (*I*^*2*^ = 20.9%, *P*_heterogeneity_ = 0.276), CC versus CT + TT model (*I*^*2*^ = 45.6%, *P*_heterogeneity_ = 0.102), and CT versus CC + TT model (*I*^*2*^ = 17.8%, *P*_heterogeneity_ = 0.298), between-study heterogeneities were determined to be significant (Table 2; Fig. 2). Moreover, there was no significant association detected for the C allele versus T allele comparison (Table 2; Fig. S4).

### 3.4 Subgroup analysis based on the characteristics of patients used as study controls

To further explore other potential sources of heterogeneity, we stratified all the studies according to attributes of study controls. In eleven studies, the participants were 2588 patients with CRC and 2912 people who were in the hospital for unrelated problems (hospital-based (HB) group), while in six studies the participants were 4541 patients with CRC and 4798 healthy individuals who were selected from the general population (population-based (PB) group). All genetic models were evaluated using the random-effect statistical model. For the PB group, the striking between-study heterogeneities were minimized. For all genetic models except for the CT versus TT model (*I*^*2*^ = 24%, *P*_heterogeneity_ = .254) and CC + CT versus TT model (*I*^*2*^ = 0, *P*_heterogeneity_ = .55), between-study heterogeneities were found not to be significant (Table 2; Fig. 3). For the HB group, there was no significant between-study heterogeneity detected (Table 2; Fig. 3; Fig. S5). Moreover, there was no significant association detected for the comparison of the C and T alleles (Table 2; Fig. S6).

### 3.5 Sensitivity analyses

The leave-one-out sensitivity analysis confirmed the robustness and reliability of our drawn conclusion. The association between SNP rs1045642 C>T and CRC remained insignificant after the removal of any included study (detailed data not shown).

### 3.6 Publication bias

Begg funnel plot and Egger test were performed to assess the publication bias. No apparent asymmetry of funnel plots was detected on visual inspection. Egger test was used to provide statistical evidence for the funnel plot. In the recessive, dominant, and overdominant models, the *P* values for Begg funnel plot were .201, .57, and .104, respectively, indicating that there was no significant publication bias (Fig. 4). There was also no publication bias in comparisons of any of the other genetic models (Fig. S7).

## 4 Discussion

Although the pathogenesis of CRC is multifactorial and mostly unclear, both internal and external factors are considered to contribute to its etiology. The multifactorial nature of the pathology of CRC calls for the quantitation of the independent risk factors. Due to the leading role of genetic factors in the pathogenesis of CRC, an in-depth understanding of the correlation of gene polymorphisms to CRC will help predict the course of the disease and take preventive measures, as well as identify potential targets for specific drug therapy. According to the findings of our present study, there was no statistically significant correlation between ABCB1 3435C>T polymorphism and the risk of CRC, regardless of the ethnicity of the study subjects and the environment of the healthy controls.

In recent years, researchers have carried out a number of studies on the association between ABCB1 gene polymorphisms and the susceptibility to various tumors. As a systematic approach that uses statistical analysis, meta-analysis is an effective way to come to conclusions about different studies that are inconsistent due to the limitations of the individual studies. A meta-analysis performed by Razi et al suggested that ABCB1 3435C>T polymorphism was not associated with the risk of multiple myeloma.^[43]^ The meta-analysis carried out by Sharif et al suggested that ABCB1 3435C>T polymorphism might be a genetic risk factor and a potential biomarker for breast cancer,^[44]^ but Tazzite et al reported that it was not associated with breast cancer risk in Morocco.^[45]^ Research by Wu et al showed that the ABCB1 C3435T polymorphism was not associated with susceptibility to gastric cancer.^[46]^ The study executed by He et al indicated that ABCB1 3435C>T polymorphism was associated with CRC risk in Asians, but Zhang et al found there was no significant association between them.^[47,48]^

In recent years, we have been concerned with the study of ABCB1 C3435T polymorphism and the risk of CRC. In the present work, 17 case-control studies were selected from Cochrane Library, PubMed, Embase databases.^[26–42]^ The results of these studies are inconsistent. Patients’ gender, age, race/ethnicity, pathological type, pathological grade, attributes of study controls, and other factors may account for inconsistencies in the results of the studies. Unfortunately, we did not get full information about patients and controls. Despite that, a comprehensive analysis was performed to identify the possible association between ABCB1 3435C>T polymorphism and CRC susceptibility. Several potential limitations can be noted in the present analysis that are inherent to any meta-analysis. First, the total sample size was not large enough, and subgroup analysis could only be performed on the basis of ethnicity and the selection of study controls. Second, only studies written in English were included in this meta-analysis. Third, some studies were of poor quality. Thus, a larger meta-analysis containing more studies, other ethnicities, and gender should be conducted in the future to improve the reliability of the conclusions.

## Acknowledgments

This work was supported by the National Natural Science Foundation of China (81903031), Zhoukou Normal University Guiding Project (ZKNUC2018011). We thank LetPub (www.letpub.com) for its linguistic assistance during the preparation of this manuscript.

## Author contributions

Guo-yin Li designed the study and drafted the initial manuscript. Li-li Han, Bai-le Zuo and Wei-liang Cai contributed to initial data analysis and interpretation. Zhen-ni Guo, Bing-hua Tong and contributed to the production of the pictures. Guo-yin Li and Zheng zhu supervised all aspects of the study, critically reviewed and revised the manuscript, and approved the final manuscript as submitted.

## Supplementary Material

Supplemental Digital Content

## Supplementary Material

Supplemental Digital Content

## Supplementary Material

Supplemental Digital Content

## Supplementary Material

Supplemental Digital Content

## Supplementary Material

Supplemental Digital Content

## Supplementary Material

Supplemental Digital Content

## Supplementary Material

Supplemental Digital Content

## Supplementary Material

Supplemental Digital Content

## References

[1] Siegel RL Miller KD Jemal A Cancer statistics, 2019. CA Cancer J Clin 2019;69:7–34. 30620402 10.3322/caac.21551

[2] Siegel RL Miller KD Fedewa SA Colorectal cancer statistics, 2017. CA Cancer J Clin 2017;67:177–93. 28248415 10.3322/caac.21395

[3] Arnold M Sierra MS Laversanne M Global patterns and trends in colorectal cancer incidence and mortality. Gut 2017;66:683–91. 26818619 10.1136/gutjnl-2015-310912

[4] Wang A Clouston SA Rubin MS Fundamental causes of colorectal cancer mortality: the implications of informational diffusion. Milbank Q 2012;90:592–618. 22985282 10.1111/j.1468-0009.2012.00675.x PMC3479384

[5] Chen W Zheng R Baade PD Cancer statistics in China, 2015. CA Cancer J Clin 2016;66:115–32. 26808342 10.3322/caac.21338

[6] Wieten E Schreuders EH Grobbee EJ Incidence of faecal occult blood test interval cancers in population-based colorectal cancer screening: a systematic review and meta-analysis. Gut 2019;68:873–81. 29934436 10.1136/gutjnl-2017-315340

[7] de Klerk OL Nolte IM Bet PM ABCB1 gene variants influence tolerance to selective serotonin reuptake inhibitors in a large sample of Dutch cases with major depressive disorder. Pharmacogenomics J 2013;13:349–53. 22641028 10.1038/tpj.2012.16

[8] Rohlff C Glazer RI Regulation of multidrug resistance through the cAMP and EGF signalling pathways. Cell Signal 1995;7:431–43. 8562304 10.1016/0898-6568(95)00018-k

[9] Ziemann C Riecke A Rudell G The role of prostaglandin E receptor-dependent signaling via cAMP in Mdr1b gene activation in primary rat hepatocyte cultures. J Pharmacol Exp Ther 2006;317:378–86. 16415092 10.1124/jpet.105.094193

[10] Pacold ME Suire S Perisic O Crystal structure and functional analysis of Ras binding to its effector phosphoinositide 3-kinase gamma. Cell 2000;103:931–43. 11136978 10.1016/s0092-8674(00)00196-3

[11] Kuo MT Liu Z Wei Y Induction of human MDR1 gene expression by 2-acetylaminofluorene is mediated by effectors of the phosphoinositide 3-kinase pathway that activate NF-kappaB signaling. Oncogene 2002;21:1945–54. 11960367 10.1038/sj.onc.1205117

[12] McCubrey JA Steelman LS Abrams SL Roles of the RAF/MEK/ERK and PI3K/PTEN/AKT pathways in malignant transformation and drug resistance. Adv Enzyme Regul 2006;46:249–79. 16854453 10.1016/j.advenzreg.2006.01.004

[13] Kaszubiak A Kupstat A Muller U Regulation of MDR1 gene expression in multidrug-resistant cancer cells is independent from YB-1. Biochem Biophys Res Commun 2007;357:295–301. 17418094 10.1016/j.bbrc.2007.03.145

[14] Oda Y Kohashi K Yamamoto H Different expression profiles of Y-box-binding protein-1 and multidrug resistance-associated proteins between alveolar and embryonal rhabdomyosarcoma. Cancer Sci 2008;99:726–32. 18377424 10.1111/j.1349-7006.2008.00748.x PMC11158972

[15] Keniry M Parsons R The role of PTEN signaling perturbations in cancer and in targeted therapy. Oncogene 2008;27:5477–85. 18794882 10.1038/onc.2008.248

[16] Lee S Choi EJ Jin C Activation of PI3K/Akt pathway by PTEN reduction and PIK3CA mRNA amplification contributes to cisplatin resistance in an ovarian cancer cell line. Gynecol Oncol 2005;97:26–34. 15790433 10.1016/j.ygyno.2004.11.051

[17] Oka M Kounoura K Narasaki F P-glycoprotein is positively correlated with p53 protein accumulation in human colorectal cancers. Jpn J Cancer Res 1997;88:738–42. 9330605 10.1111/j.1349-7006.1997.tb00445.x PMC5921503

[18] Rimler A Jockers R Lupowitz Z Differential effects of melatonin and its downstream effector PKCalpha on subcellular localization of RGS proteins. J Pineal Res 2006;40:144–52. 16441551 10.1111/j.1600-079X.2005.00290.x

[19] Sui H Fan ZZ Li Q Signal transduction pathways and transcriptional mechanisms of ABCB1/Pgp-mediated multiple drug resistance in human cancer cells. J Int Med Res 2012;40:426–35. 22613403 10.1177/147323001204000204

[20] Wang YJ Zhang YK Zhang GN Regorafenib overcomes chemotherapeutic multidrug resistance mediated by ABCB1 transporter in colorectal cancer: in vitro and in vivo study. Cancer Lett 2017;396:145–54. 28302530 10.1016/j.canlet.2017.03.011 PMC5507680

[21] Brambila-Tapia AJ MDR1 (ABCB1) polymorphisms: functional effects and clinical implications. Rev Invest Clin 2013;65:445–54. 24687344

[22] Fung KL Gottesman MM A synonymous polymorphism in a common MDR1 (ABCB1) haplotype shapes protein function. Biochim Biophys Acta 2009;1794:860–71. 19285158 10.1016/j.bbapap.2009.02.014 PMC2810319

[23] Wang H Cao H Xu Z SNP rs2596542G>A in MICA is associated with risk of hepatocellular carcinoma: a meta-analysis. Bioscience Rep 2019;39:BSR20181400. 10.1042/BSR20181400 PMC6504665 30967497

[24] DerSimonian R Laird N Meta-analysis in clinical trials. Control Clin Trials 1986;7:177–88. 3802833 10.1016/0197-2456(86)90046-2

[25] Mantel N Haenszel W Statistical aspects of the analysis of data from retrospective studies of disease. J Natl Cancer Inst 1959;22:719–48. 13655060

[26] Kurzawski M Drozdzik M Suchy J Polymorphism in the P-glycoprotein drug transporter MDR1 gene in colon cancer patients. Eur J Clin Pharmacol 2005;61:389–94. 15912392 10.1007/s00228-005-0926-5

[27] Lee BI Choi KY Lee KM [Is C3435T polymorphism of MDR1 related to inflammatory bowel disease or colorectal cancer in Korean?]. Korean J Gastroenterol 2006;47:22–9. 16434865

[28] Komoto C Nakamura T Sakaeda T MDR1 haplotype frequencies in Japanese and Caucasian, and in Japanese patients with colorectal cancer and esophageal cancer. Drug Metab Pharmacokinet 2006;21:126–32. 16702732 10.2133/dmpk.21.126

[29] Bae SY Choi SK Kim KR Effects of genetic polymorphisms of MDR1, FMO3 and CYP1A2 on susceptibility to colorectal cancer in Koreans. Cancer Sci 2006;97:774–9. 16800822 10.1111/j.1349-7006.2006.00241.x PMC11160064

[30] Osswald E Johne A Laschinski G Association of MDR1 genotypes with susceptibility to colorectal cancer in older non-smokers. Eur J Clin Pharmacol 2007;63:9–16. 17146660 10.1007/s00228-006-0225-9

[31] Potocnik U Glavac D Dean M Common germline MDR1/ABCB1 functional polymorphisms and haplotypes modify susceptibility to colorectal cancers with high microsatellite instability. Cancer Genet Cytogenet 2008;183:28–34. 18474294 10.1016/j.cancergencyto.2008.01.023

[32] Petrova DT Nedeva P Maslyankov S No association between MDR1 (ABCB1) 2677G&gt;T and 3435C&gt;T polymorphism and sporadic colorectal cancer among Bulgarian patients. J Cancer Res Clin Oncol 2008;134:317–22. 17674045 10.1007/s00432-007-0279-9 PMC12161662

[33] Panczyk M Balcerczak E Piaskowski S ABCB1 gene polymorphisms and haplotype analysis in colorectal cancer. Int J Colorectal Dis 2009;24:895–905. 19415305 10.1007/s00384-009-0724-0

[34] Andersen V Ostergaard M Christensen J Polymorphisms in the xenobiotic transporter Multidrug Resistance 1 (MDR1) and interaction with meat intake in relation to risk of colorectal cancer in a Danish prospective case-cohort study. Bmc Cancer 2009;9:407. 19930591 10.1186/1471-2407-9-407 PMC2797527

[35] Khedri A Nejat-Shokouhi A Salek R Association of the colorectal cancer and MDR1 gene polymorphism in an Iranian population. Mol Biol Rep 2011;38:2939–43. 20127181 10.1007/s11033-010-9957-9

[36] Sainz J Rudolph A Hein R Association of genetic polymorphisms in ESR2, HSD17B1, ABCB1, and SHBG genes with colorectal cancer risk. Endocr Relat Cancer 2011;18:265–76. 21317201 10.1530/ERC-10-0264

[37] Campa D Sainz J Pardini B A comprehensive investigation on common polymorphisms in the MDR1/ABCB1 transporter gene and susceptibility to colorectal cancer. Plos One 2012;7:e32784. 22396794 10.1371/journal.pone.0032784 PMC3292569

[38] Kim H Um J Kim Y Association of a multidrug resistance 1 gene polymorphism and colorectal cancer in the Korean population. Orient Pharm Exp Med 2013;13:225–30.

[39] Wu H Kang H Liu Y Association ofABCB1 genetic polymorphisms with susceptibility to colorectal cancer and therapeutic prognosis. Pharmacogenomics 2013;14:897–911. 23746184 10.2217/pgs.13.78

[40] Ozhan G Kara M Sari FM Associations between the functional polymorphisms in the ABCB1 transporter gene and colorectal cancer risk: a case-control study in Turkish population. Toxicol Mech Methods 2013;23:235–9. 23193993 10.3109/15376516.2012.743639

[41] Stanko G Kaminski M Bogacz A The importance of G2677T/A and C3435T polymorphisms of the MDR1 gene in the aetiology of colorectal cancer. Prz Gastroenterol 2016;11:35–40. 27110309 10.5114/pg.2015.51185 PMC4814531

[42] Wang F Huang Z Zheng K Two SNPs of ATP-binding cassette B1 gene on the risk and prognosis of colorectal cancer. Int J Clin Exp Pathol 2015;8:3083–9. 26045821 PMC4440130

[43] Razi B Anani SG Omidkhoda A Multidrug resistance 1 (MDR1/ABCB1) gene polymorphism (rs1045642 C &gt; T) and susceptibility to multiple myeloma: a systematic review and meta-analysis. Hematology 2018;23:456–62. 29495954 10.1080/10245332.2018.1443897

[44] Sharif A Kheirkhah D Reza SM ABCB1-C3435T polymorphism and breast cancer risk: a case-control study and a meta-analysis. J Buon 2016;21:1433–41. 28039704

[45] Tazzite A Kassogue Y Diakite B Association between ABCB1 C3435T polymorphism and breast cancer risk: a Moroccan case-control study and meta-analysis. BMC Genet 2016;17:126. 27580695 10.1186/s12863-016-0434-x PMC5007843

[46] Wu DD Zhang JX Li J Lack of association of the MDR1 C3435T polymorphism with susceptibility to gastric cancer and peptic ulcer: a systemic review and meta-analysis. Asian Pac J Cancer Prev 2014;15:3021–7. 24815441 10.7314/apjcp.2014.15.7.3021

[47] He T Mo A Zhang K ABCB1/MDR1 gene polymorphism and colorectal cancer risk: a meta-analysis of case-control studies. Colorectal Dis 2013;15:12–8. 23279665 10.1111/j.1463-1318.2012.02919.x

[48] Zhang D Wang C Zhou Z Meta-Analysis of ABCB1 3435C&gt; T Polymorphism and Colorectal Cancer. Pak J Med Sci 2013;29:1269–74. 24353734 10.12669/pjms.295.3758 PMC3858949

