# Supplementary material for: Association between ABCB1 (3435C>T) polymorphism and susceptibility of colorectal cancer: A meta-analysis
Source: Medicine (Baltimore). 2020 Feb 21;99(8):e19189. doi: 10.1097/MD.0000000000019189 (PMC7034701; doi:10.1097/MD.0000000000019189)
Supplement: Supplemental Digital Content [file medi-99-e19189-s001.doc]

| Supplemental Table 1 Score of quality assessment | |
| --- | --- |
| **Criteria** | **Score** |
| Representativeness of case |  |
| Selected from population cancer registry | 2 |
| Selected from hospital | 1 |
| No method of selection described | 0 |
| Representativeness of control |  |
| Population-based | 3 |
| Blood donors | 2 |
| Hospital-based | 1 |
| Not described | 0 |
| Ascertainment of NHL case |  |
| Histopathologic confirmation | 2 |
| by patient medical record | 1 |
| Not described | 0 |
| Control selection |  |
| Controls matched with cases by age and sex | 2 |
| Controls matched with cases only by age or by sex | 1 |
| Not matched or not descried | 0 |
| Genotyping examination |  |
| Genotyping done blindly and quality control | 2 |
| Only genotyping done blindly or quality control | 1 |
| Unblinded and without quality control | 0 |
| HWE |  |
| HWE in the control group | 1 |
| HWD in the control group or not mentioned | 0 |
| Total sample size |  |
| > 1000 | 3 |
| 501 - 1000 | 2 |
| 201 - 500 | 1 |
| ≤ 200 | 0 |
