# Supplementary figures and images for: Association between ABCB1 (3435C>T) polymorphism and susceptibility of colorectal cancer: A meta-analysis
Source: Medicine (Baltimore). 2020 Feb 21;99(8):e19189. doi: 10.1097/MD.0000000000019189 (PMC7034701; doi:10.1097/MD.0000000000019189)

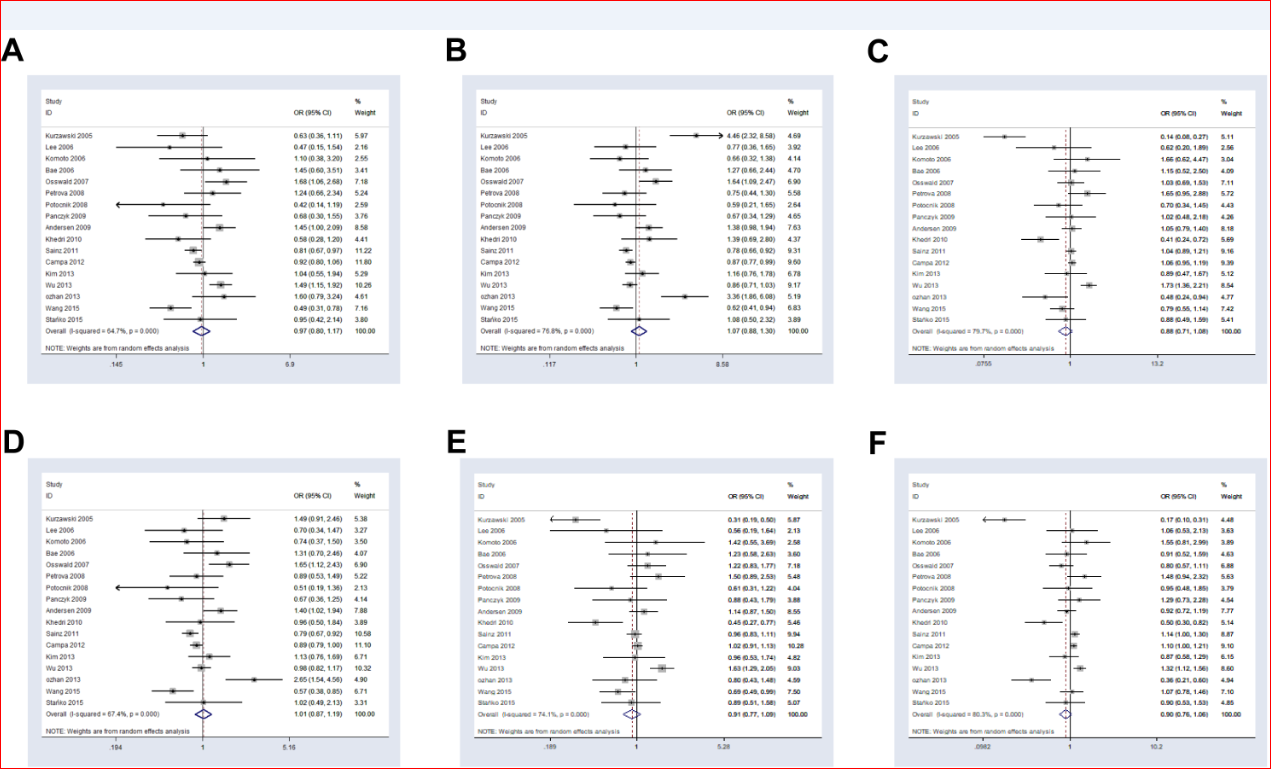

Supplement: Supplemental Digital Content [file medi-99-e19189-s002.doc]

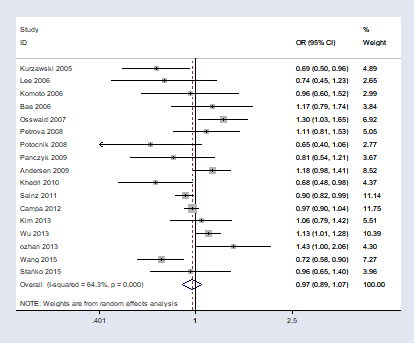

Supplement: Supplemental Digital Content [file medi-99-e19189-s003.tif]

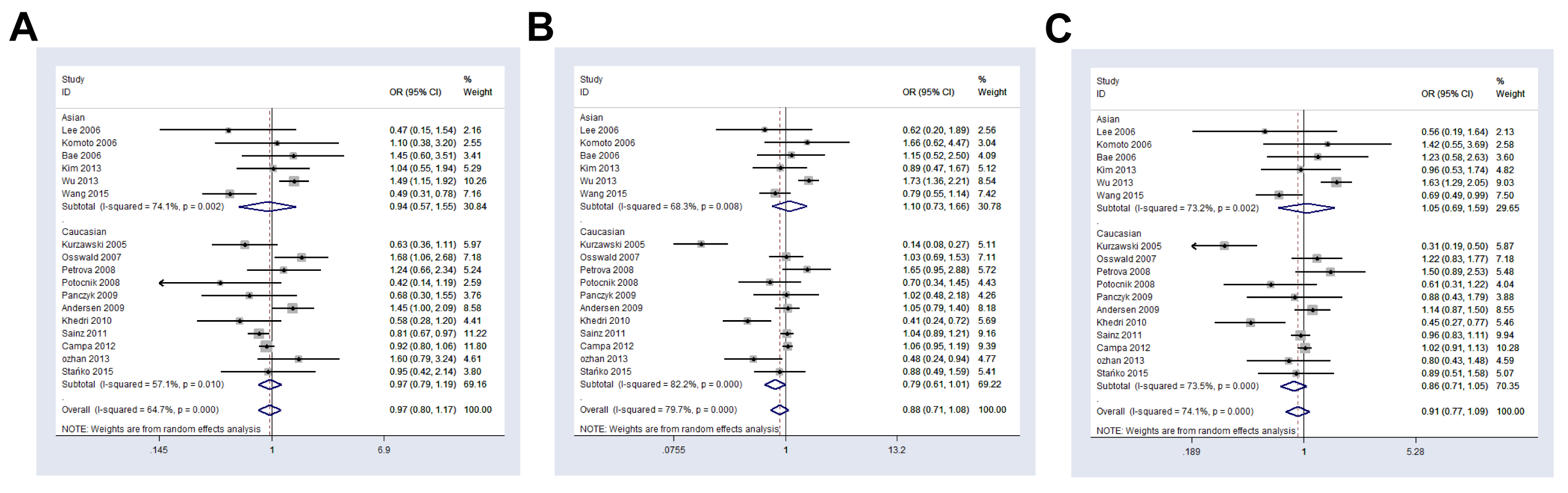

Supplement: Supplemental Digital Content [file medi-99-e19189-s004.tif]

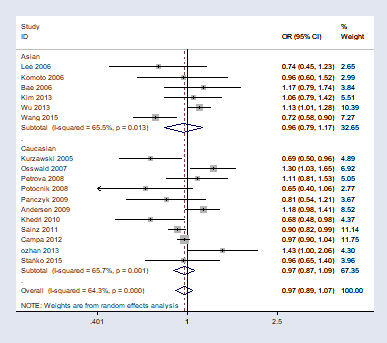

Supplement: Supplemental Digital Content [file medi-99-e19189-s005.tif]

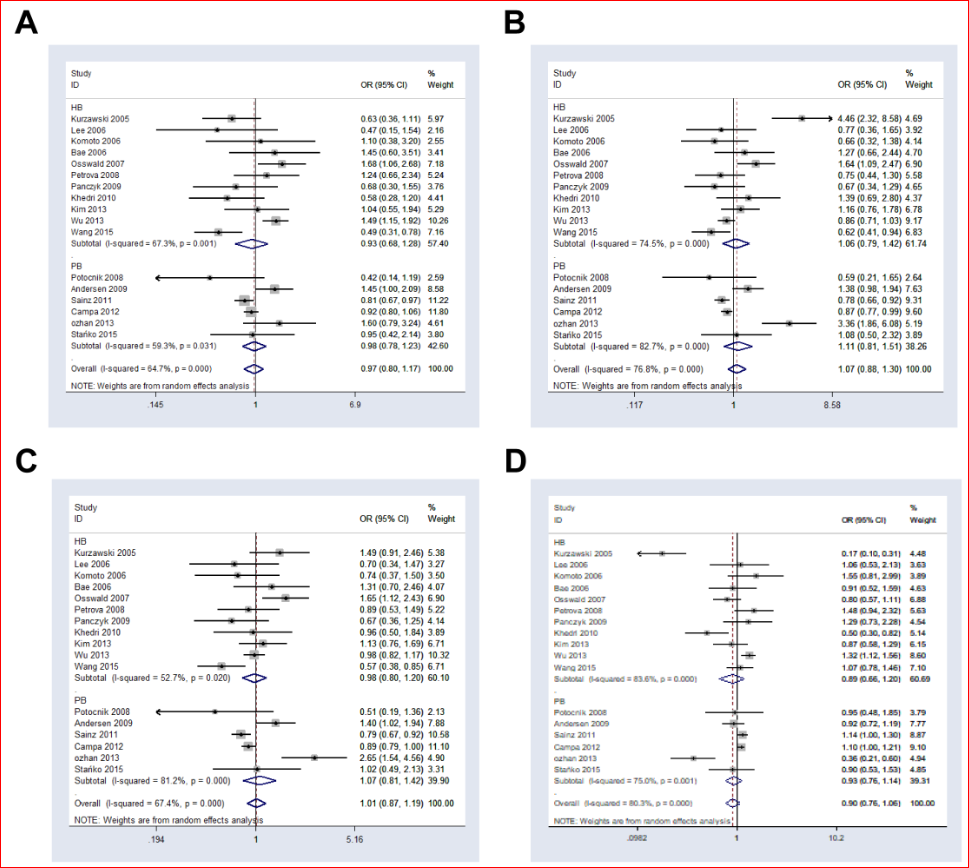

Supplement: Supplemental Digital Content [file medi-99-e19189-s006.doc]

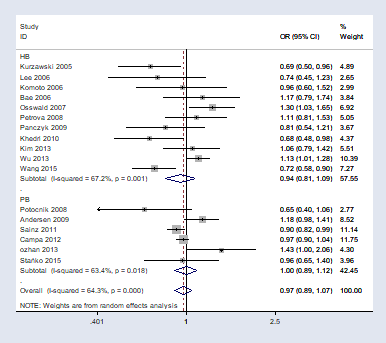

Supplement: Supplemental Digital Content [file medi-99-e19189-s007.tif]

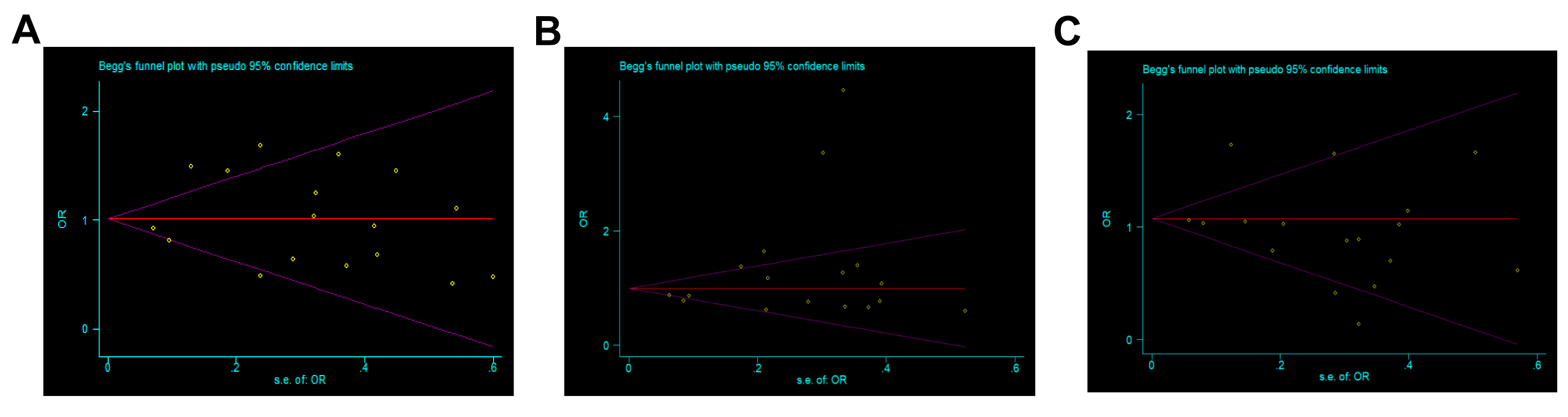

Supplement: Supplemental Digital Content [file medi-99-e19189-s008.tif]
